# Changes in Prefrontal Cortex–Thalamic Circuitry after Acoustic Trauma

**DOI:** 10.3390/biomedicines9010077

**Published:** 2021-01-14

**Authors:** Kristin M. Barry, Donald Robertson, Wilhelmina H. A. M. Mulders

**Affiliations:** The Auditory Laboratory, School of Human Sciences, University of Western Australia, 35 Stirling Highway, Crawley, WA 6009, Australia; Kristin.Barry@anu.edu.au (K.M.B.); Don.Robertson@uwa.edu.au (D.R.)

**Keywords:** acoustic trauma, medial geniculate nucleus, prefrontal cortex, electrophysiology

## Abstract

In the adult auditory system, loss of input resulting from peripheral deafferentation is well known to lead to plasticity in the central nervous system, manifested as reorganization of cortical maps and altered activity throughout the central auditory pathways. The auditory system also has strong afferent and efferent connections with cortico-limbic circuitry including the prefrontal cortex and the question arises whether this circuitry is also affected by loss of peripheral input. Recent studies in our laboratory showed that PFC activation can modulate activity of the auditory thalamus or medial geniculate nucleus (MGN) in normal hearing rats. In addition, we have shown in rats that cochlear trauma resulted in altered spontaneous burst firing in MGN. However, whether the PFC influence on MGN is changed after cochlear trauma is unknown. We investigated the effects of electrical stimulation of PFC on single neuron activity in the MGN in anaesthetized Wistar rats 2 weeks after acoustic trauma or sham surgery. Electrical stimulation of PFC showed a variety of effects in MGN neurons both in sham and acoustic trauma groups but inhibitory responses were significantly larger in the acoustic trauma animals. These results suggest an alteration in functional connectivity between PFC and MGN after cochlear trauma. This change may be a compensatory mechanism increasing sensory gating after the development of altered spontaneous activity in MGN, to prevent altered activity reaching the cortex and conscious perception.

## 1. Introduction

Loss of input from peripheral deafferentation in adult sensory systems is well known to lead to plasticity in the central nervous system, leading to reorganization of cortical maps [1,2,3,4,5] and increases in cortical spontaneous activity [6,7]. Changes are not restricted to cortical areas but also extend to thalamus [8,9,10,11] and even lower levels of the sensory pathways, as has been extensively documented in the auditory system following trauma to the cochlea and consequent hearing loss [12,13,14,15].

The auditory system, just like all other sensory systems, has elaborate afferent and efferent connections with brain structures involved in emotional and memory processing such as the prefrontal cortex (PFC), amygdala, and hippocampus [16,17] and this cortico-limbic brain circuitry is thought to contribute to the conscious perception of auditory information [18]. Projections from PFC to the auditory thalamus (medial geniculate nucleus (MGN)) are thought to be indirect and inhibitory, via the predominantly GABA-ergic thalamic reticular nucleus [19,20,21]. Inputs to the thalamic reticular nucleus may be involved in the process of sensory gating, controlling sensory attention by modifying afferent information en route to the cortex [22,23,24].

The question then arises whether this circuitry is affected by loss of input. Indeed, human imaging data have shown structural and functional changes in this cortico-limbic and auditory brain circuitry following hearing impairment. Hearing loss has been shown to be associated with significantly lower grey matter volume in the PFC in individuals with hearing loss compared to normal-hearing individuals [25,26]. Increased functional coupling between the PFC and auditory cortex has been demonstrated in hearing impaired individuals compared to normal hearing controls [27]. In addition, there is evidence that sensory gating can be negatively affected by loss of peripheral input in the auditory system. Hearing impairments are associated with a higher risk of auditory hallucinations [28] and phantom auditory perceptions such as tinnitus [29,30]. Interestingly, higher levels of co-activation of the thalamus and the executive network, which includes PFC, have been observed in first episode schizophrenics with auditory verbal hallucinations [31].

In the present study, we investigated whether cochlear trauma affects the functionality of the pathways between PFC and MGN, by measuring the effects of PFC electrical stimulation on the firing rates of single neurons in MGN in Wistar rats with and without cochlear trauma from a prior acoustic over-exposure. In our laboratory, we have developed a rat model of cochlear trauma that results in a temporary hearing loss and the development of tinnitus in about 50% of animals. In this model, we have shown plastic changes in MGN burst firing parameters in all animals independent of whether they developed tinnitus [10]. These plastic changes are therefore most likely to be due to the cochlear trauma and subsequent loss of peripheral input even in the absence of a shift in peripheral thresholds (so-called hidden hearing loss [32,33]). We have also already demonstrated functional connectivity between PFC and MGN in rats, using single neuron recordings in MGN whilst electrically stimulating the PFC [34].

## 2. Materials and Methods

### 2.1. Animals

Eight male Wistar rats, weighing between 285–588 g (mean 417.8 g), were used. Experimental protocols complied with the Code of Practice of the National Health and Medical Research Council of Australia and were approved by the Animal Ethics Committee of the University of Western Australia.

### 2.2. Recovery Procedure for Acoustic Trauma and Sham

All animals underwent a recovery procedure and were anaesthetised with 5% isoflurane and maintained with 1.5–2.5% isoflurane for the duration of the procedure. When deep anaesthesia was obtained, as determined by the absence of the foot withdrawal reflex, animals were placed on a heating blanket in a soundproof room and mounted in hollow ear bars. Subcutaneous electrodes were placed in the fore and hind paw of the animal to monitor the animal’s ECG. The ear bar contralateral to the acoustic stimulus was blocked with plasticine and animals were either exposed to an AT (continuous loud tone for 2 h, 10 kHz, 124 dB SPL to the unblocked ear (procedure ear)) (*n* = 4) or not exposed (sham; *n* = 4). The sham animals were kept under anaesthesia for the same period as the AT animals. Animals recovered for 2 weeks then underwent a non-recovery electrophysiological experiment.

### 2.3. Anaesthesia for Non-Recovery Electrophysiological Experiment

The procedures for anaesthesia induction and maintenance for final non-recovery electrophysiology experiments (single neuron recordings) were as described in detail in previous studies from our laboratory [10,34,35]. Anaesthesia was induced by intraperitoneal injection of urethane (1.3 g/kg). Ten minutes later, animals received a subcutaneous injection of 0.05 mL atropine sulfate (0.05 mg/mL) and an intramuscular injection of 0.1 mL Hypnorm (0.315 mg/mL fentanyl citrate and 10 mg/mL fluanisone). Some animals required an additional dose of 0.1 mL Hypnorm to maintain deep anaesthesia. When full depth of anaesthesia was reached as assessed by the absence of foot withdrawal in response to foot pinch, animals were placed on a heating blanket in a soundproof room. Rectal temperature was checked every hour and maintained at 37.5 °C. Animals received a tracheostomy and a plastic tracheal cannula was inserted for artificial ventilation later during the experiment. Animals’ ECG was measured as described above and they were mounted in a stereotaxic frame using hollow ear bars. The head was levelled and a partial craniotomy was performed using a small dental drill at identified coordinates [36]. This allowed access to prefrontal cortex (PFC) and medial geniculate nucleus (MGN). Animals also received an intramuscular injection of 0.1 mL pancuronium bromide (2 mg/mL) 15 min before data collection and they were artificially ventilated on carbogen (95% O_2_ and 5% CO_2_). Animals required a further intramuscular injection of 0.1 mL pancuronium bromide every 2 h to maintain full paralysis. The requirement was indicated during the experiment by the observation that the animals’ breathing was no longer dependent on the ventilator, suggesting paralysis was wearing off. In order to assess the animals’ level of analgesia ECG in response to foot pinch was noted every hour. No effects on ECG were observed throughout the experiments.

### 2.4. Single Neuron Recordings in Medial Geniculate Nucleus

For single neuron recordings, sound stimuli were delivered to the procedure ear (AT or sham) while electrophysiological recordings were made in the contralateral MGN. The non-procedure ear was blocked with plasticine. All sound stimuli were presented in a calibrated sound system through a ½″ condenser microphone driven in reverse as a speaker (Brüel & Kjær, type 4134, Nærum, Denmark) and were synthesized by a computer using custom software (Neurosound MI Lloyd) and a DIGI 96 soundcard connected to an analog/digital interface (ADI-9 DS, RME Intelligent Audio Solution, Haimhausen, germany). Sample rate was 96 kHz. The sound system was calibrated using an ⅛” microphone (Brüel & Kjær, type 2670, Nærum, Denmark) in place of the animal’s eardrum and a calibrated sound source (Brüel & Kjær, type 4231, Nærum, Denmark) to measure the output of the sound system (dB SPL re 20 µPa).

Noise stimuli (50-ms duration, 1 ms rise/fall times) were used as a search stimulus for single neurons in MGN. Single neuron recordings were obtained using a tungsten in glass microelectrode [37] or glass insulated platinum iridium electrode (Frederick Haer & Co, Bowdoin, USA). Analysed data were recorded from neurons that showed spikes which were clearly distinguishable and well isolated from background electrical activity for the duration of experiments. When a single neuron was isolated, its characteristic frequency (CF) and acoustic threshold at CF were determined audio-visually as described previously when recording from guinea pig inferior colliculus neurons and rat MGN neurons in our laboratory [10,34,38]. The spontaneous firing rate was then measured for a period of 10 s while input to the speaker was turned off to eliminate the possibility of a low-level background noise emanating from the sound system.

For electrical stimulation of PFC a custom-made bipolar tungsten electrode connected to an A-M Systems Isolated Pulse Stimulator (Model 2100) was placed in PFC as described previously [34,39]. The timing of electrical stimuli was controlled by the Neurosound software. Electrical stimuli were delivered as shock trains (pulse duration 0.5 ms, train duration 50 ms, rate 200 Hz). Maximum current (1 mA) was applied to increase the likelihood of seeing an effect of stimulation in the MGN. From these experiments, histograms of 500 ms samples of firing rate with and without PFC electrical stimulation were obtained (75 sweeps) to assess the effects of brief repetitive electrical stimulation on MGN single neuron firing rate.

Additionally, histograms of a single 60 s sample of spontaneous firing rate before and after 2 min of electrical stimulation of PFC on stimulated firing rate were obtained from these animals to assess the effect of prolonged electrical stimulation. For individual neurons, the change in firing rate (stimulated firing rate–spontaneous firing rate) caused by the stimulation was calculated and if the change was >10% it was categorized as showing an effect in response to PFC stimulation as has been done previously by our laboratory [34].

For group comparisons, the average change in firing rate per bin in the histogram was calculated for the sham and AT groups in both the brief repetitive (bin size 1 ms) and prolonged electrical stimulation experiments (bin size 1 s). The total change was then compared using a Mann–Whitney U Test. After obtaining data on brief repetitive and prolonged PFC electrical stimulation animals were euthanized with an intraperitoneal injection of 0.3 mL of Lethabarb.

### 2.5. Statistical Analysis

As data was not normally distributed non-parametrical statistical analysis was performed. A Wilcoxon signed-rank was used within sham and AT groups to assess if there was an effect before and after PFC electrical stimulation. To analyse the distribution of neurons to PFC responses, a Chi-squared Test was done on the number of neurons classified as showing which response type.

## 3. Results

### 3.1. Single Neuron Data

Single neuron recordings were obtained two weeks after sham or AT surgery. Placement of MGN recording electrodes was confirmed by electrophysiological recordings showing clear noise-induced cluster activity, as described in previous publications from our laboratory [10,34,35]. Stimulating electrodes were positioned in prelimbic PFC [40] as we have shown previously that activation of this part of PFC results in modulation of firing rates in MGN [34].

Spontaneous firing rates, characteristic frequency (CF, the frequency to which a neuron shows the lowest threshold) and threshold were obtained from single neurons (53 MGN neurons in the sham group and 42 MGN neurons in the acoustic trauma (AT) group). We also collected time-histograms of supra-threshold sound-evoked responses for cell classification [41,42]. In the sham animals, neuronal CF varied from 120 Hz to 44 kHz (mean 13.8 ± 1.89 kHz) and these were not significantly different (Mann–Whitney U test) from the AT animals, which had CFs which varied from 150 Hz to 41 kHz (mean 12.2 ± 1.59 kHz). Sham thresholds at CF varied from 17 to 92 dB SPL (mean 46 dB SPL) and were not significantly different (Mann–Whitney U test) from the AT animals which had thresholds from 17 to 94 dB SPL (mean 52 dB SPL). In sham animals, 44 (83%) of these neurons showed onset characteristics to sound, one (2%) a sustained response, four (8%) neurons showed an offset response, two (4%) neurons were found to have on and off response to sound, and two (4%) were found to be unresponsive (no evoked response) to sound. Thirty-three (79%) of these neurons in AT animals showed onset characteristics to sound, whereas six (14%) neurons were found to have a sustained response to sound and three (7%) neurons showed an offset response.

### 3.2. Effects of Brief Repetitive PFC Electrical Stimulation

**Figure 1:**
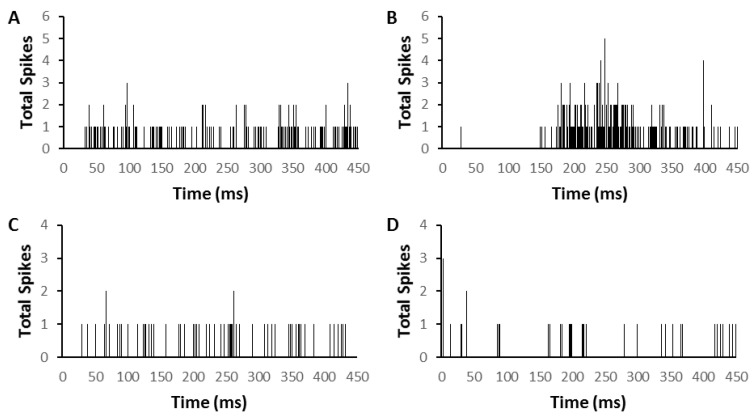
Time histograms (75 sweeps) showing two examples of medial geniculate nucleus (MGN) neuron firing in silence before (left column, (**A**,**C**)) and after (right column (**B**,**D**)) brief repetitive electrical stimulation of prefrontal cortex (PFC) (pulse duration 0.5 ms, train duration 50 ms, rate 200 Hz). (**A**,**B**) Onset response neuron (CF = 2.8 kHz; threshold = 30 dB) showing increase in firing after PFC electrical stimulation. (**C**,**D**) Onset response neuron (CF = 8.1 kHz; threshold = 78 dB) showing decrease increase in firing after PFC electrical stimulation. The time point of 0 denotes the end of electrical stimulus in (**B**,**D**).

Time histograms (500 ms) recorded in silence immediately before and then after brief repetitive (50 ms) electrical stimulation of PFC were obtained from 50 of the 53 MGN neurons in the sham group and 26 of the 42 MGN neurons in the AT group (examples shown in Figure 1).

In the sham group, 28 (56%) of these 50 neurons showed a decrease in firing rate in response to PFC electrical stimulation (negative change > 10%), with a mean decrease of 56.4 ± 4.7%. Eleven (22%) of the 50 neurons showed an increase in firing rate (positive change > 10%), with a mean increase of 88.5 ± 28.7% and the remaining 11 (22%) neurons collected from the sham group showed no change in firing rate in response to PFC brief repetitive electrical stimulation. Similarly, 15 (58%) of the 26 neurons from the AT group were categorized as showing a decrease in response to PFC electrical stimulation (62.6 ± 7.4%), 7 (27%) showed an increase in firing rate, with a mean increase of 76.75 ± 22.40% and the remaining and 4 (15%) neurons showed no change in firing rate.

Chi-square analysis of the proportions of neuronal responses (increase, decrease or no response) to brief PFC stimulation revealed no significant differences between the AT and sham groups. In addition, a Mann–Whitney U test revealed no significant changes between the groups in the percentage of increased or decreased firing rate after electrical stimulation.

**Figure 2:**
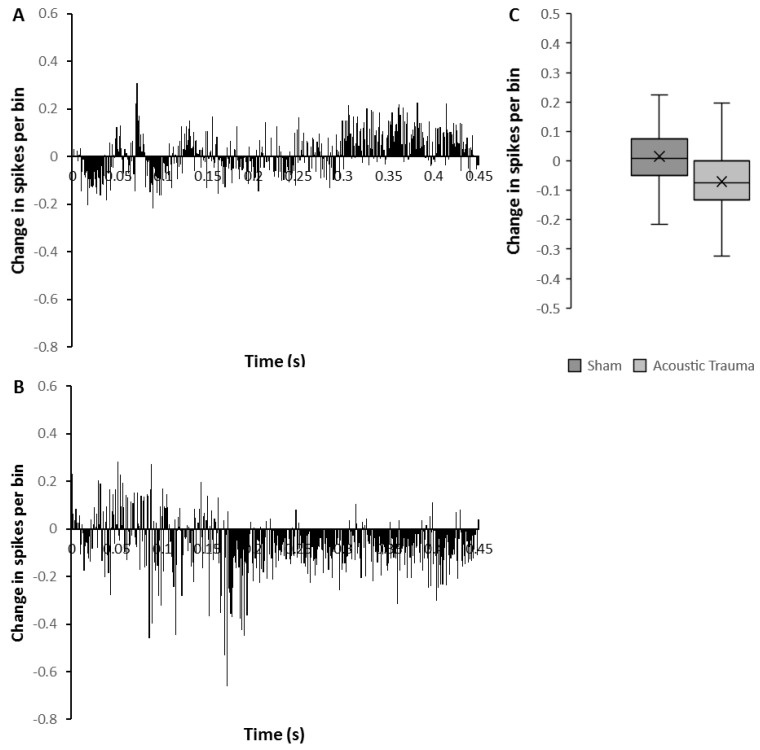
Changes in medial geniculate nucleus (MGN) firing after acoustic trauma (AT) and with prefrontal cortex (PFC) brief repetitive electrical stimulation. (**A**,**B**) Time histograms showing temporal pattern of mean change (spikes/bin over 450 ms following 50 ms of electrical stimulation) in medial geniculate nucleus (MGN) firing rate with PFC repetitive electrical stimulation 2 weeks after sham (**A**) or acoustic trauma (**B**). (**C**) The average change of spikes per bin after PFC electrical stimulation in sham versus AT animals. The time point of 0 denotes the end of electrical stimulus in (**A**,**B**).

The mean firing rate of the sham group before PFC electrical stimulation was 2.90 ± 0.003 spikes/s (*n* = 50) and this was significantly increased (7%; Mann–Whitney U test *p* < 0.0001) following PFC electrical stimulation (3.10 ± 0.003 spikes/s). However, in contrast, the mean firing rate in the AT group (*n* = 26) showed a marked and significant reduction after PFC electrical stimulation from 2.52 ± 0.005 spikes/s to 1.59 ± 0.004 spikes/s (decrease of 37%; Mann–Whitney U test *p* < 0.0001). To investigate detailed the temporal pattern of change, the average amount of change over time was calculated from the histograms before and after brief repetitive PFC electrical stimulation (Figure 2). The temporal pattern of change was clearly different in AT animals (Figure 2B) compared to the sham animals (Figure 2A). In the AT group, there was a marked decrease of firing rate starting approximately 170 ms after stimulation had ceased and this decrease lasted for the remainder of the recording (Figure 2B). In contrast, sham animals showed substantial enhancement of firing rate starting approximately 300 ms from the end of stimulation (Figure 2A). The amount of change in firing rate overall reflected the temporal pattern, showing a significant reduction in the AT group and an increase in firing rate in the sham group (Figure 2C, Mann–Whitney U test, *p* = 0.0001).

### 3.3. Effects of Prolonged PFC Electrical Stimulation

**Figure 3:**
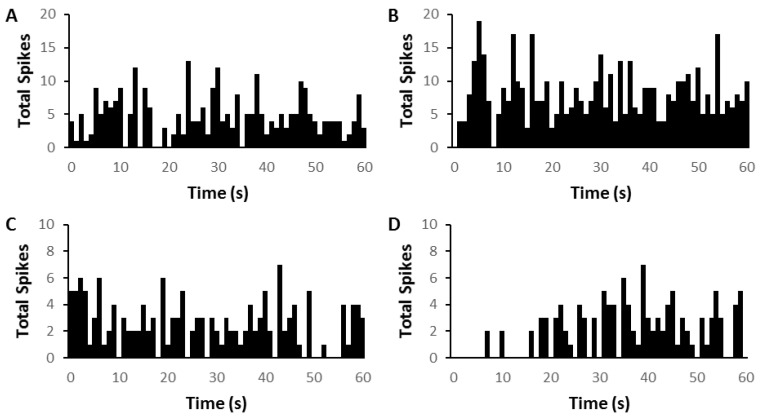
Time histograms showing two examples of medial geniculate nucleus (MGN) neuron firing in silence before (left column, (**A**,**C**) and after (right column (**B**,**D**)) prolonged electrical stimulation of prefrontal cortex (PFC). (**A**,**B**) Onset response neuron(CF = 6.9 kHz; threshold = 37 dB) showing an immediate increase in firing followed by a general increase in overall firing after PFC electrical stimulation (**C**,**D**) Onset response neuron (CF = 10.5 kHz; threshold = 32 dB) showing decrease in firing after PFC electrical stimulation. Electrical stimulation by shock trains (pulse duration 0.5 ms, train duration 2 min, rate 200 Hz).

From the same eight animals (four AT and four sham) that were used for the brief repetitive PFC electrical stimulation experiment, the firing rates of MGN neurons in silence were also measured 1 min before and after a prolonged 2-min electrical stimulation of PFC. Examples are shown in Figure 3. Data were obtained from 48 MGN neurons in the sham group and 35 MGN neurons in the AT group. Of the 48 neurons from the sham group, 16 (33%) of the showed a decrease in firing rate (mean = 39.42%), 20 (42%) had an elevation of firing rate (mean of 72.12%), and the remaining 12 (25%) showed no change in firing rate in response to prolonged PFC electrical stimulation. In the 35 AT group neurons, 14 (40%) showed a decrease in firing rate (61.84%) and this decrease was significantly larger as compared to the decrease in sham animals (Mann–Whitney U test, *p* = 0.03). Seventeen (49%) of the 35 neurons from the AT group showed a mean increase in firing rate of 106.07% but this difference was not significant. The remaining 4 (11%) AT group neurons showed no change in firing rate. Chi-square analysis showed no differences in these proportions of neuronal responses to prolonged PFC stimulation between the groups.

Similar to what was observed after the brief repetitive PFC electrical stimulation, the average absolute amount of change was greater in AT animals compared to sham animals and resulted in a significant reduction rather than increase of firing (Mann–Whitney U test, *p* = 0.0001). In the AT group, mean spontaneous firing rate before PFC electrical stimulation was 2.78 ± 0.09 spikes/s and this became significantly reduced after prolonged PFC electrical stimulation to 2.32 ± 0.08 spikes/s (a decrease of 17%; Mann–Whitney U test, *p* = 0.0001). In the sham group, mean spontaneous firing rate was 2.52 ± 0.37 spikes/s and this was significantly increased to 3.08 ± 0.05 spikes/s (an increase of 22%; Mann–Whitney U test, *p* = 0.0001) after PFC electrical stimulation.

**Figure 4:**
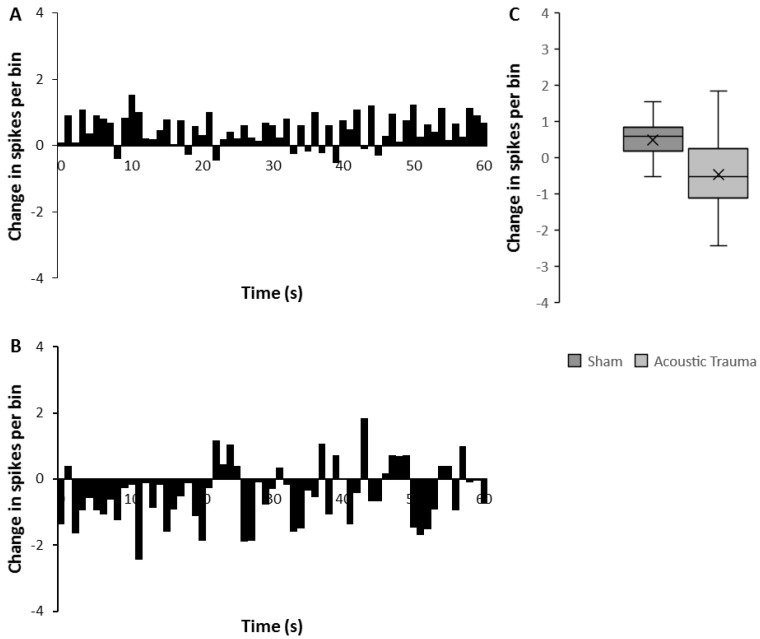
Changes in medial geniculate nucleus (MGN) firing after acoustic trauma (AT) and with prefrontal cortex (PFC) prolonged electrical stimulation. (**A**,**B**) Rate histograms showing mean change (spikes/bin over 60 s following 2 min of PFC electrical stimulation) in medial geniculate nucleus (MGN) firing rates, 2 weeks after sham (**A**) or acoustic trauma (**B**). (**C**) The average change of spikes per bin after PFC electrical stimulation in sham versus AT animals. The time point of 0 denotes the end of stimulus in (**A**,**B**).

A similar temporal analysis to that performed for the effects of brief electrical stimulation (Figure 4) clearly reflected the overall increase in mean firing rate in the sham group and this increase persisted for the full 60 s recorded (Figure 4A,C). In contrast, AT animals showed an overall decrease in firing rate following the prolonged PFC stimulation, a response that was significantly different from that observed in the sham animals (Figure 4C, Mann–Whitney U test *p* < 0.0001). This decrease was marked and consistent in the first 20 s and then showed some fluctuations in the remaining 40 s of recording (Figure 4B).

Data from both the brief repetitive and prolonged electrical stimulation PFC experiments were compared to assess if they were any relationships between individual responses to brief repetitive and prolonged PFC electrical stimulation. Data are summarized in Table 1. From the sham group, 48 neurons were assessed using both the brief repetitive and prolonged stimulation paradigm. Twenty-six (54%) of these 48 neurons showed a response to both brief repetitive and prolonged electrical PFC stimulation. Of these 26, 11 (43%) neurons showed the same response (either increase, decrease or no change) to brief repetitive and prolonged electrical PFC stimulation. The other 15 (57%) neurons showed different responses to brief repetitive and prolonged electrical PFC stimulation. From the AT group, data following both stimulation paradigms were obtained from 26 neurons. Eighteen (72%) of these 26 showed a response to both brief repetitive and prolonged electrical PFC stimulation. Of these 18 neurons, 10 (56%) neurons showed the same response to brief repetitive and prolonged electrical PFC stimulation. The other eight (44%) showed different responses. These results suggest that there is no clear relationship between neuronal firing rate response to brief repetitive and prolonged PFC electrical stimulation as effects observed in response to brief repetitive stimulation do not predict effects observed with prolonged stimulation.

## 4. Discussion

This paper provides the first evidence in an animal model for altered functional connectivity between PFC and MGN after cochlear trauma. The overall effect of PFC stimulation on MGN changed from an excitatory effect in sham animals to an inhibitory effect at 2 weeks after a cochlear trauma.

We have previously shown that an AT paradigm as used in this study, causes immediate cochlear damage, indicated by a temporary threshold shift as measured using the auditory brainstem response [10]. The absence of a permanent threshold shift in cochlear thresholds however, does not imply that cochlear function is unchanged after the recovery of thresholds, as numerous previous studies have shown that acoustic trauma, even in the absence of a threshold change, causes considerable loss of auditory nerve fibres [33,43,44]. In addition, we have shown that this particular AT paradigm results in altered spontaneous firing in MGN [10], in line with numerous other studies showing alterations in spontaneous firing along the auditory pathway after AT [12,13,45,46,47].

Electrical stimulation of PFC resulted in diverse changes to the firing rate of individual MGN neurons in agreement with our previously published data in animals without exposure to an AT [34]. The diversity of MGN responses to PFC electrical stimulation is unlikely to be due to recording from different MGN subdivisions, CFs or cell types as we have previously found no relationship of sound response type or subdivision on responses to PFC electrical stimulation [34]. The diverse responses may be due to the fact that PFC has multiple indirect pathways to the thalamic reticular nucleus, which projects to MGN [21,48]. The thalamic reticular nucleus, although predominantly GABA-ergic [24], is known to elicit inhibitory and excitatory responses in the MGN, which may provide an explanation for the varied MGN neuronal responses obtained in this study [22].

After the AT, this diversity in responses to PFC stimulation could still be observed, and no significant change was observed in the proportion of excitatory or inhibitory responses compared to the sham animals. However, the average effect caused by PFC electrical stimulation changed from an overall mild excitation in the sham animals to significant overall inhibition of firing rates in the AT animals. This effect was observed both with brief repetitive stimulation as well as prolonged stimulation of PFC. In view of the fact that the relative proportions of neurons showing excitation or inhibition did not change after AT this result suggests that the inhibitory effects after AT increased in magnitude. GABAergic synapses can be strengthened by inhibitory long-term potentiation (iLTP), induced by the clustering of GABA receptors [49,50]. Such iLTP may be the result of further homeostatic plasticity mechanisms within MGN in response to changes in spontaneous firing following the acoustic trauma [10] and would potentially change the response to activation of the thalamic reticular nucleus, the prominent relay for all pathways en route from PFC to MGN [21,48]. Loss of peripheral input in the auditory system drives homeostatic plasticity and results in increased gain within the auditory pathway and an associated increase in spontaneous firing [15,47,51], due to increased expression of excitatory receptors, such as those for glutamate and reduced expression of inhibitory receptors (GABA and glycine) [52,53,54].

The implications of these findings for auditory perception and the possible development of tinnitus will need further study. The changes we have observed may represent an early compensatory effect to prevent altered spontaneous firing in MGN [10] to reach the cortex and lead to conscious perception. This would be in line with a role of the PFC in sensory gating of non-salient auditory information [20,23,29,30]. Conceivably, variations in the amount of increased inhibition of PCF on MGN could play a role in the individual variation in susceptibility to tinnitus development [55]. However, most animal models using similar cochlear trauma show behavioural evidence of tinnitus becoming apparent only after at least 4 to 6 weeks [56,57,58,59] and the present results were obtained only two weeks after cochlear trauma. Furthermore, we did not investigate the presence of tinnitus in this study. Our study captured a single time-point at which we know spontaneous firing in MGN is altered [10] and where we have now shown changes in functional PFC circuity. It is possible that the alterations observed in this study are transient and may differ in both their nature and magnitude at longer recovery times. Indeed, it has been suggested that the breakdown of sensory gating at the level of MGN would lead to development of tinnitus [29,30,60]. In addition, all experiments were carried out under general anaesthesia and although the results provide unequivocal evidence for a change in the pathways after AT, they leave open the question of precisely how they function and how they affect auditory perception in the awake state. Finally, the findings of this study could represent an important mechanism for preventing maladaptive neural activity from generating phantom auditory sensations in the form of tinnitus.

## Figures and Tables

**Table 1:** Responses of individual medial geniculate nucleus neurons that were assessed using both brief repetitive and prolonged electrical stimulation of the PFC in sham and AT animals, showing the variability of responses to either type of electrical stimulation within neurons. Shown are absolute numbers (percentage).

		Brief Repetitive PFC Stimulation					
		Increase		Decrease		No Effect	
**Prolonged** **PFC** **stimulation**		Sham	AT	Sham	AT	Sham	AT
	Increase	4 (20%)	5 (33%)	11 (55%)	7 (47%)	5 (25%)	3 (20%)
	Decrease	4 (25%)	1 (6%)	7 (44%)	5 (63%)	5 (31%)	2 (25%)
	No Effect	2 (17%)	1 (33%)	8	2 (66%)	2 (17%)	0 (0%)

## Data Availability

Data available on request.

## Abbreviations

ABR	Auditory brainstem response
AT	Acoustic trauma
CF	Characteristic frequency
MGN	Medial geniculate nucleus
PFC	Prefrontal cortex
PB	Phosphate buffer
PSTH	Peristimulus time histogram
PTS	Permanent threshold shift
